# Outcomes of Coronary Artery Bypass Graft Surgery in Africa: A Systematic Review and Meta-Analysis

**DOI:** 10.7759/cureus.47541

**Published:** 2023-10-23

**Authors:** Oluwanifemi O Akintoye, Oyinlola P Fasina, Tijani S Adiat, Promise U Nwosu, Mohammed O Olubodun, Bukola G Adu

**Affiliations:** 1 Cardiothoracic Surgery, Surgery Interest Group of Africa, Lagos, NGA

**Keywords:** cardiac surgery, africa, cardiac surgery in africa, meta-analysis, systematic review, cabg, coronary artery bypass graft

## Abstract

Coronary artery bypass graft (CABG) surgery has been in practice for many decades, and it is one of the most commonly performed cardiac surgeries worldwide. While there are several studies reporting data on perioperative outcomes following CABG in developed countries, there is a staggering paucity of data and evidence reporting the outcomes in developing areas such as Africa. Thus, it is important to study the practice and outcome of CABG in Africa to establish its clinical efficacy and safety in this region and identify factors that might be limiting its practice. The overall aim of this study is to identify all relevant clinical data on CABG in Africa and report on the perioperative outcomes and practice of CABG in the African population. Electronic search was performed using three online databases, PubMed, African Journal Online, and Research Gate, from inception to June 2023. The preferred reporting items for systematic reviews and meta-analysis (PRISMA) guideline was utilised for this study. Relevant studies fulfilling predefined eligibility criteria were included in the study. Intraoperative details, such as the number of grafts performed, operative, bypass, and cross-clamp time, were reported. The primary endpoint assessed were early mortality and overall mortality. The secondary endpoints included length of hospital stay, intensive care unit stay, and postoperative complications, such as renal impairment, atrial fibrillation, and surgical site infection. The data were pooled together and meta-analyzed using a random effect model for proportions and mean for meta-analysis with R software (version 4.3.1 (2023-06-16); R Development Core Team, Vienna, Austria). This systematic review identified 42 studies that fulfilled the study eligibility criteria, including 21 randomised controlled trials, 20 observational studies, and one cross-sectional study. Only four out of the 54 countries in Africa had studies carried out that met the criteria for this review; they included Algeria, Egypt, Nigeria, and South Africa, with a majority from Egypt. Meta-analysis reported a pooled early mortality and pooled overall mortality of 3.51% and 3.73%, respectively, for the total cohort of patients. The result of this meta-analysis suggests that mortality outcomes following CABG in Africa are relatively higher than those in developed nations. Several issues, such as lack of financial resources and poor infrastructure, continue to hinder the optimal practice of CABG procedures in many parts of Africa. Further studies focused on finding factors associated with outcomes following CABG should be done. Though there were a few limitations to the study largely from a lack of data from several regions and countries in Africa, the result from this meta-analysis can serve as a benchmark for future studies until more relevant data are reported.

## Introduction and background

In the last few decades, urbanisation and westernisation have led to an epidemiological transition in the prevalence of diseases in developing regions, such as Africa. Africa has experienced a decline in the incidence of infectious diseases and an exponential surge in cardiovascular diseases, including coronary artery disease (CAD) [1]. Earlier studies suggested that the prevalence and death rates from CAD were low in the black African population. However, more recent reports suggest an increase in the incidence and prevalence of CAD in Africa [2,3].

Coronary artery bypass graft (CABG) is the conventional surgical revascularisation method for treating for severe CAD as they have been shown to significantly relieve patient symptoms [4]. CABG surgery entails rerouting blood around diseased or narrowed coronary vessels to improve blood supply to the myocardium distal to them [5].

With conventional CABG being associated with many post-operative complications, such as wound infections, renal dysfunction, and excessive bleeding [6-8], surgical advancement over the years has led to the development of less invasive CABG techniques, such as minimally invasive CABG and robotic CABG in developed regions [4], thus reducing the incidence of these post-operative adverse outcomes. 

However, in many countries in Africa, the practice of CABG is still quite limited. While there are several studies reporting data on perioperative outcomes following CABG in developed countries, there is a staggering paucity of data and evidence reporting these perioperative outcomes in Africa. Though Africa's population is almost 20% of the world's population, reports have shown that Africa contributes less than 0.3% of the global research papers on cardiovascular health [9].

Due to the expensive costs of cardiac surgeries, many African countries, which are low- and middle-income economies, lack the necessary healthcare funds to establish and maintain robust cardiac centres [10]. This underdeveloped healthcare system inadvertently leads to poorer clinical surgical outcomes, including CABG. Thus, given this limiting factor to optimal healthcare, it is important to study the practice and outcomes of CABG in the African population to establish its clinical efficacy in Africa. The primary aim of this study is to analyse and report on the perioperative outcomes following CABG in the African population. The two main endpoints of interest are early mortality and overall mortality. For our study, we defined early mortality as any mortality reported as either in-hospital mortality, 30-day mortality, or early mortality. We defined overall mortality as all deaths occurring from any cause. 

## Review

Methods

In conducting this review, we utilised the preferred reporting system for systematic review and meta-analysis (PRISMA) guidelines. A comprehensive literature search was done using PubMed, African Journal Online, and Research Gate to identify relevant papers from inception till June 2023. The search strategy employed for the study was jointly devised by all authors and is summarised in Table 1. The search results were exported into rayyan.ai (a systematic review software; Rayyan Systems, Cambridge, MA) for detailed screening and deduplication. To ensure consistency and accuracy, each paper was screened twice by two independent authors, and conflicts were resolved by consensus following discussion with other authors. Titles and abstracts were initially screened, after which full-text screening was carried out.

**Table 1:** Search strategies on the electronic databases. CABG - Coronary artery bypass graft

Database	MesH terms/Keywords	Hits
PubMed	("coronary artery bypass"[MeSH Terms] OR ("coronary"[All Fields] AND "artery"[All Fields] AND "bypass"[All Fields]) OR "coronary artery bypass"[All Fields]) AND ("africa"[MeSH Terms] OR "africa"[All Fields] OR "africa s"[All Fields] OR "africas"[All Fields] OR ("saharan"[All Fields] OR "saharans"[All Fields]) OR (Each of the 54 African countries mentioned individually)	645
PubMed	coronary artery bypass AND Africa	126
PubMed	CABG AND Africa	35
PubMed	coronary artery bypass OR CABG AND outcome AND Africa	55
PubMed	coronary artery bypass OR CABG AND complication AND Africa	48
PubMed	Coronary artery bypass OR CABG AND (each of the 54 African countries)	645
African Journal Online	Coronary artery bypass graft OR CABG	130
Research Gate	Coronary artery bypass graft OR CABG AND Africa	100 (first ten pages)

Eligibility Criteria

All original research papers, including controlled trials and retrospective and prospective studies, on adults who underwent CABG in an African country and reported specific clinical outcomes following CABG in any of the 54 African countries were considered eligible for this study. All papers that reported mortality outcomes as either overall mortality, in-hospital mortality, and 30-day mortality following CABG were included in the study.

Exclusion Criteria 

Case reports, case series, systematic reviews, meta-analyses, abstracts, and commentaries were excluded from the study. Papers not written in the English language were also excluded from the study. Studies that reported a combined outcome of patients for CABG and other coronary revascularisation methods (i.e., percutaneous and studies not carried out on the human population) were also excluded from the study. All papers that did not report on any of the three interesting mortality outcomes, overall mortality, in-hospital mortality, and 30-day mortality following CABG were excluded from the study.

Data Extraction and Variables of Interest 

Summary statistics of the following variables of interest were extracted from the included research articles into an Excel sheet: author, year of publication, country, study design, follow-up period, age, gender, comorbidities (hypertension, diabetes mellitus, dyslipidaemia, smoking, atrial fibrillation, renal dysfunction), Euro-score, New York Heart Association (NYHA) class, and left ventricular ejection fraction (LVEF). Perioperative data extracted included operative time, aortic cross-clamp time, bypass time, and the mean number of grafts.

Outcomes of Interest

Postoperative data on length of intensive care unit (ICU) stay, length of hospital stay, 30-day mortality, in-hospital mortality, overall mortality, and post-operative complications, such as renal dysfunction, atrial fibrillation, surgical site infection, pulmonary, and bleeding, were also extracted. 

For our study, the pooled early mortality outcome was reported to include data extracted as either in-hospital mortality, 30-day mortality, or early mortality, while overall mortality included mortality rates and death rates reported from all included studies.

Statistical Analysis 

The baseline demographic characteristics of the total cohort were summarised using the appropriate summary statistics and shown using tables. The continuous variables were summarised using mean, while categorical variables were summarised using counts and proportions. For the meta-analysis, raw data on post-operative outcomes were pooled together using a random effect model for proportions and mean to produce a single pooled incidence value for outcomes of interest.

All statistical analysis was done using R software (version 4.3.1 (2023-06-16) -- "Beagle Scouts"; R Development Core Team, Vienna, Austria). 

Results

Distribution and Types of Studies

**Figure 1:**
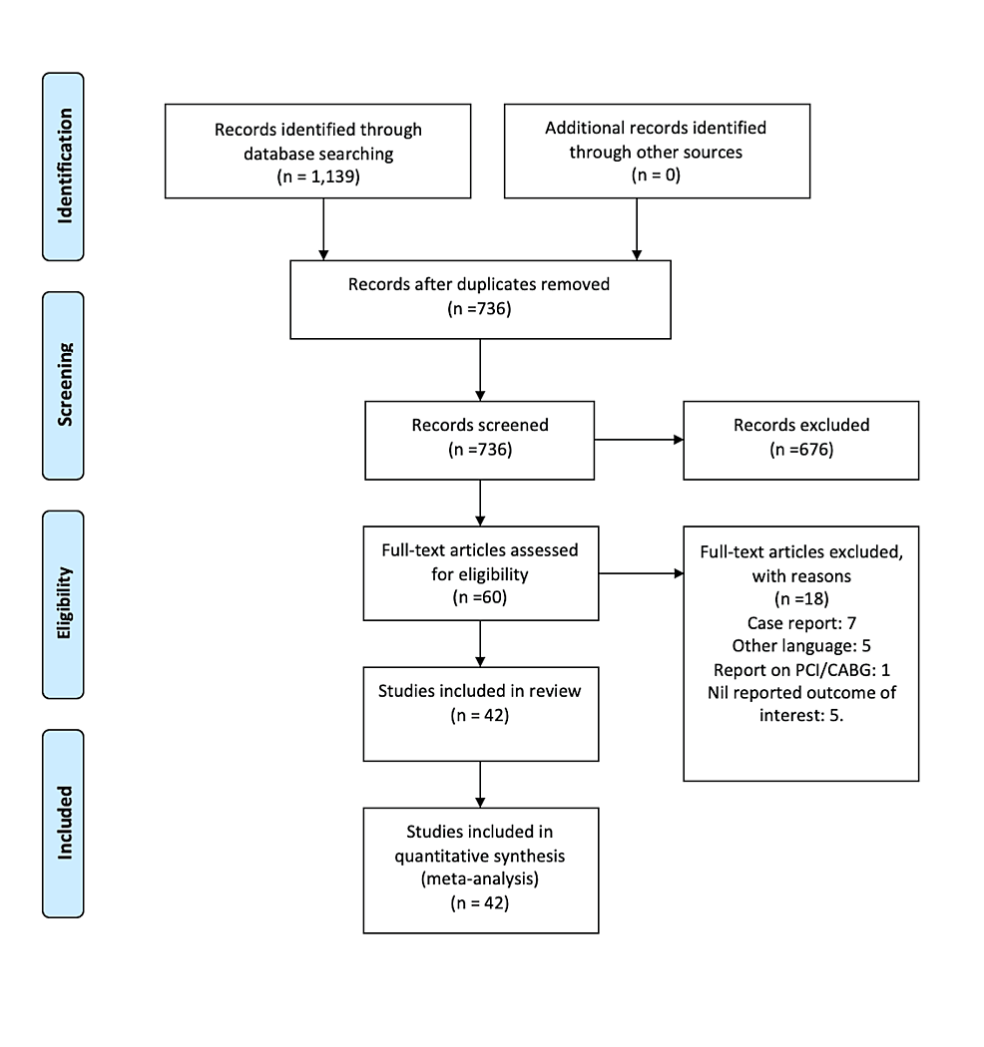
Flowchart showing the steps in the selection of studies for this systematic review. AJOL - African Journal Online; PCI - Percutaneous coronary intervention; CABG - Coronary artery bypass graft

An initial total of 1,139 papers were identified following a search of the electronic databases. A total of 403 papers were identified as duplicates and excluded. Of the 736 papers remaining, 676 papers were excluded following the screening of titles and abstracts. Subsequently, an additional 18 papers were excluded for not meeting our criteria during full-text screening. A total of 42 papers met the predefined eligibility criteria for our study and were included in this systematic review and meta-analysis [11-52]. Five papers were excluded because of language not in English, 11 because of the wrong study population or study design (e.g., case reports), and two papers were excluded due to a lack of access to full text. The PRISMA flowchart showing the screening process is presented in Figure 1.

Of the 42 included papers, 21 were randomized trials, 20 were observational (13 prospective and 7 retrospective), and one was a cross-sectional study. Only four of the 54 African countries had studies that met our eligibility criteria. Most 31 (73.8%) of the studies were from Egypt, and the three other African countries with studies included were South Africa (9), Nigeria (1), and Algeria (1). Table 2 shows the characteristics of the studies included in this systematic review and meta-analysis.

**Table 2:** Characteristics of the papers included in the review. RCT - Randomised controlled trials

Author	Country	Year of Publication	Study Design	Sample size
Yasser et al. [34]	Egypt	2009	RCT	100
McFarlane et al. [37]	South Africa	2007	RCT	142
Abdou et al. [20]	Egypt	2018	Prospective	552
Swart et al. [17]	South Africa	2016	Retrospective	1750
Swart et al [12]	South Africa	2010	Retrospective	451
Abdelwahab et al. [45]	Egypt	2018	RCT	42
Swart et al. [16]	South Africa	2012	Retrospective	873
Kamel et al. [52]	Egypt	2021	Prospective	80
Shokri et al. [51]	Egypt	2019	RCT	286
Ammar et al. [50]	Egypt	2018	RCT	60
Boukhmis et al. [49]	Algeria	2022	Prospective	235
Singab [48]	Egypt	2020	Prospective	168
Abdel-Salam et al. [47]	Egypt	2016	Prospective	740
Wolmarans et al. [46]	South Africa	2021	Retrospective	100
Alsadeq et al. [11]	Egypt	2021	RCT	68
El-Morsy et al. [13]	Egypt	2012	RCT	50
Soliman et al. [14]	Egypt	2022	RCT	186
Kahil et al. [15]	Egypt	2012	RCT	50
Ngcobo et al. [18]	South Africa	2020	Cross-sectional	28
Abdelrazek et al. [19]	Egypt	2021	Prospective	89
Mogahd et al. [21]	Egypt	2017	RCT	70
Reichie et al. [27]	South Africa	2021	Retrospective	1,218
Omar et al. [26]	Egypt	2021	Prospective	1,000
Osinaike et al. [24]	Nigeria	2015	Retrospective	194
Elmarsafawi et al. [33]	Egypt	2016	RCT	94
Turky et al. [35]	Egypt	2017	RCT	40
Manie et al. [25]	South Africa	2008	Prospective	245
Mohamed et al. [42]	Egypt	2020	Prospective	100
EI Gindy et al. [36]	Egypt	2022	Prospective	249
Khallaf et al. [41]	Egypt	2019	RCT	60
Harris et al. [23]	South Africa	2009	Prospective and Retrospective	55
El-Haddad et al. [22]	Egypt	2012	RCT	100
Shokri et al. [30]	Egypt	2022	RCT	80
Rezk et al. [28]	Egypt	2020	RCT	100
Ibraheem et al. [38]	Egypt	2019	Retrospective	16
Amr et al. [43]	Egypt	2010	RCT	45
Fawzy et al. [44]	Egypt	2015	RCT	200
Ramadan et al. [31]	Egypt	2020	Prospective	150
Omar et al. [39]	Egypt	2020	RCT	279
Hamed et al. [40]	Egypt	2018	RCT	60
Ramadan et al. [32]	Egypt	2018	Prospective	80
Mubarak et al. [29]	Egypt	2021	RCT	100

Demographic Characteristics

The total number of patients included in the meta-analysis is 10,585 patients. A summary of available demographics is provided in Table 3. The average age of the total cohort was 58.35 years. The majority of the patients (73.8%) were males, 64.2% had hypertension, 45% had diabetes mellitus, 48.2% had a smoking history, 67.3% had dyslipidaemia, and 21.9% (286/1,301) had preoperative atrial fibrillation.

**Table 3:** Preoperative characteristics of study participants. n = total number of individuals with characteristics N = total population of which the presence or absence of characteristics is reported % or mean = continuous variables reported as mean and categorical variables as percentages

Characteristics	n/N	% or mean
Age (years)		58.35
Male	7,814/10,585	73.8%
Female	2,771/10,585	26.2%
Hypertension	2,273/3,540	64.2%
Diabetes Mellitus	4,056/9,022	45.0%
Dyslipidaemia	1,485/2,208	67.3%
Smoking	3,459/7,181	48.2%
Atrial fibrillation	286/1,301	22.0%
Renal dysfunction	658/3,668	17.9%
LVEF (average) cm		55.57
LVEF <40%	168/3,403	4.0%
LVEF >40%	3,235/3,403	95.1%
NYHA I-II	574/1,085	52.9%
NYHA III-IV	511/1,085	47.1%
Euro SCORE		4.12

The majority (95.1%) of the patients had LVEF of >40%, with an average LVEF of 53.58% across the cohort. Additionally, a larger proportion (50.8%) of patients had an NYHA III and below classification. The average operative time, cardiopulmonary bypass time, and aortic cross-clamp time were 254.11 minutes, 112.98 minutes, and 67.3 minutes, respectively, as shown in Table 4.

**Table 4:** Operative characteristics of study participants. n = counts of operative characteristics N = total population in which operative characteristic is reported % or mean = continuous variables are reported as mean and categorical as percentage

Characteristics	n/N	% or mean
Elective surgeries	1,193/1,882	64.9%
Emergency surgeries	689/1,838	
Mean no of graft		2.6
Arterial graft	1,711/1,895	90.3%
CPB (min)		111.98
Cross clamp time (min)		67.30
Operative time (min)		254.11

Post-operative Outcomes

**Figure 2:**
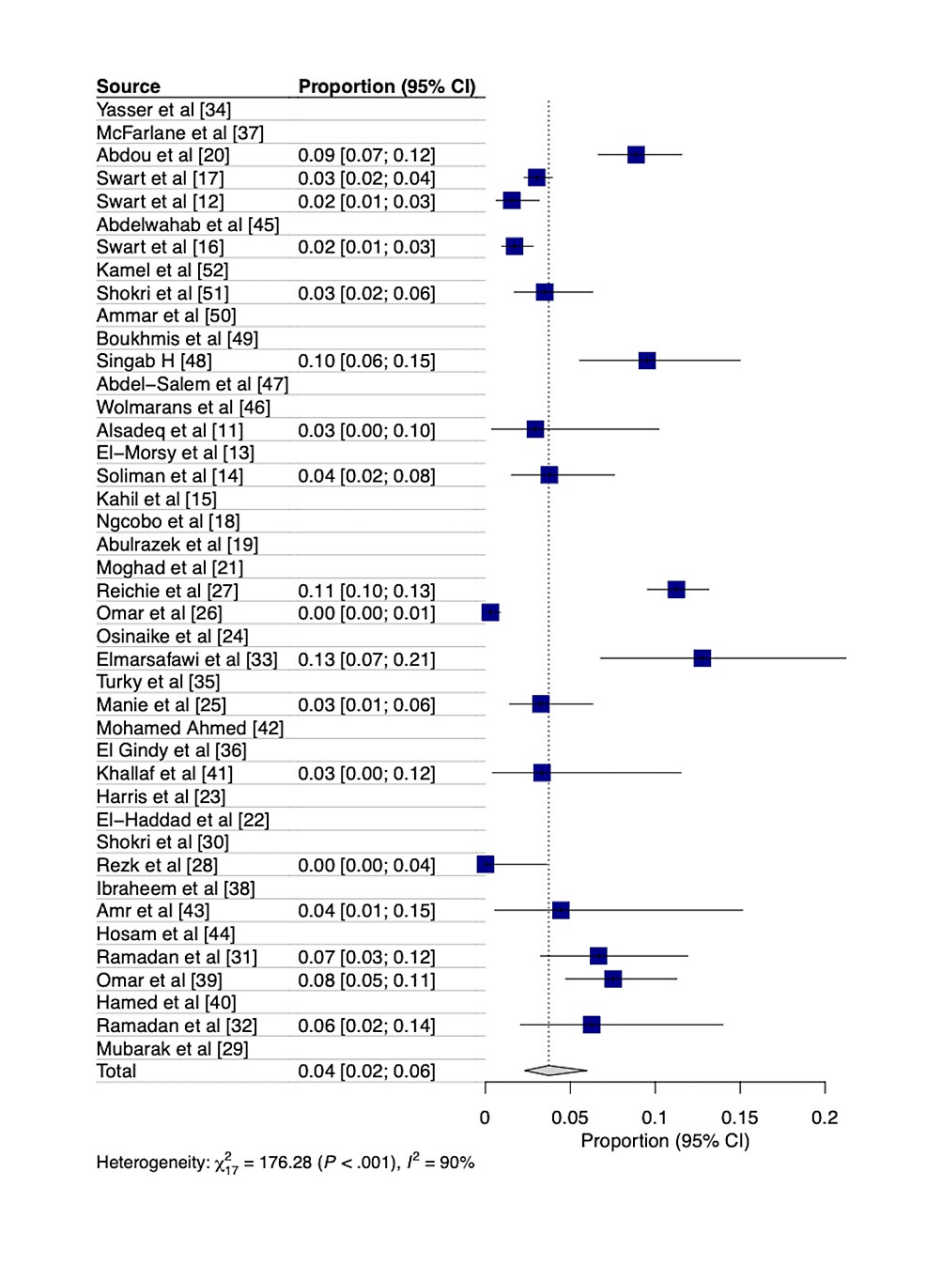
Forest plot showing the pooled overall mortality following coronary artery bypass graft surgery in Africa. 95% CI = 95% Confidence interval Seventeen of the 42 papers reported on overall mortality and were included in this analysis.

**Figure 3:**
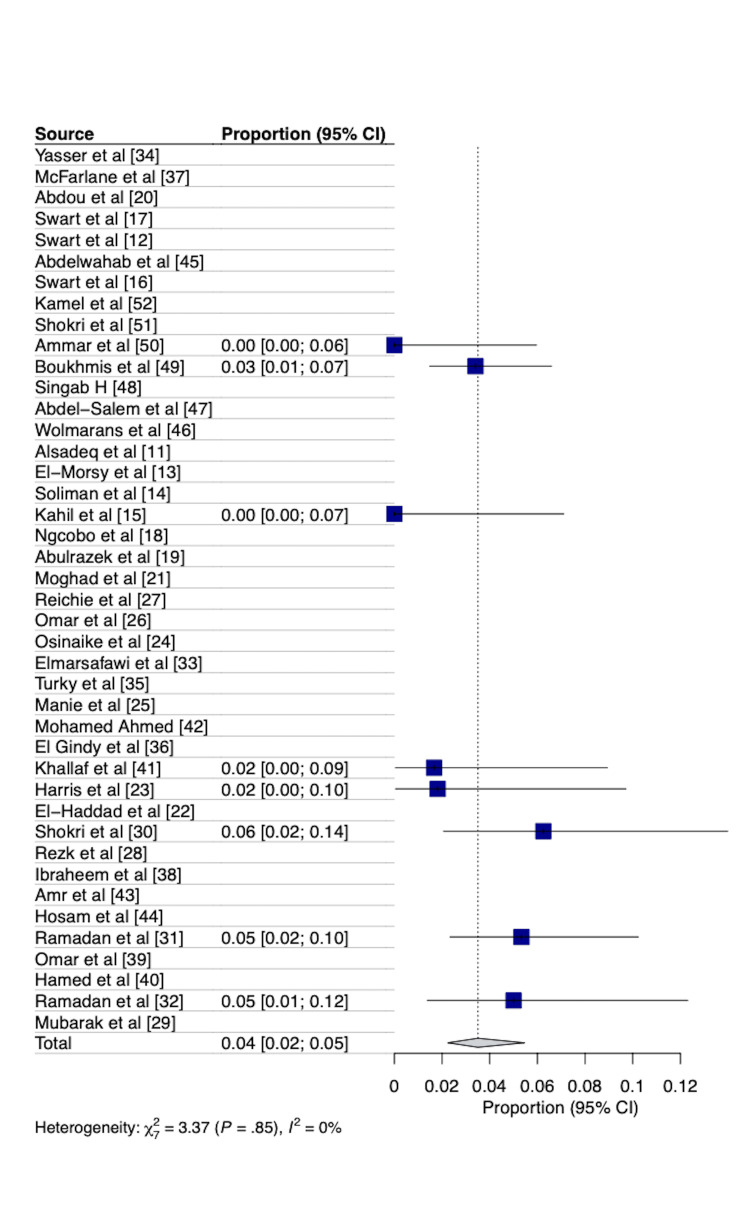
Forest plot showing the pooled early mortality following coronary artery bypass graft surgery in Africa. 95% CI = 95% Confidence interval Only eight of the 42 papers reported on early mortality (30-day, in-hospital, or early mortality) and were included in this analysis.

Meta-analysis reported a pooled overall mortality rate of 3.73% (95% CI=2.30­-5.98) and an early mortality rate of 3.51% (95% CI=2.24-5.46) in the cohort of patients, as shown in Figures 2, 3. A total of 17 papers reported on overall mortality and were included in the analysis for the pooled mortality, while only eight papers reported on early mortality (30-day, in-hospital, or early).

The pooled prevalence of all analysed postoperative outcomes and complications are summarised in Table 5. The rate of surgical site infection was 4.59% (95% CI=1.67-1.20), post-operative atrial fibrillation was 16.25% (95% CI=11.0-23.3), and post-operative renal dysfunction was 5.38% (95% CI=2.86-9.89). The pooled incidence of respiratory complications, including chest infection, pleural effusion, atelectasis, pneumothorax, respiratory failure, and pulmonary oedema, was 16.45% (95% CI=4.86-43.13). Respiratory complications accounted for one of the outcomes with the highest incidence. Significant post-operative bleeding occurred in 6.23% (95% CI=1.45-2.31%), with 5.36% (95% CI=2.54-1.09) of the patients reporting a return to theatre for exploration and control of bleeding. Only 2.38% (95% CI=1.43-3.93) and of the patients developed post-operative myocardial infarction. More than one-tenth of the patients 10.25% (95% CI=2.12-3.76) required post-operative mechanical cardiovascular support by way of an intra-aortic balloon pump.

**Table 5:** Pooled post-operative outcomes. n = count of individuals with outcome N = total population size of which outcome is reported % or mean = Continuous variables reported as mean and categorical variables as percentages 95% CI = 95% Confidence interval IABP =Intra‐aortic balloon pump counterpulsation LVEF = Left ventricular ejection fraction

Characteristics	n/N	% or mean	Pooled prevalence (95% CI)
Overall mortality	359/7,605	4.7%	3.73 (2.30­-5.98)
Early mortality	27/220	3.5%	3.51 (2.24-5.46]
Surgical site infection	281/5,310	5.3%	4.59 (1.67-1.20)
Renal dysfunction	209/4,031	5.2%	5.38 (2.86-9.89]
Atrial fibrillation	492/3,532	13.9%	16.25 (11.0-23.3)
Post-op myocardial infarction	53/658	8.1%	7.82 (5.03-1.20]
Post-op stroke	82/4,390	1.9%	2.38 (1.43-3.93]
Post-op bleeding	271/3,236	8.4%	6.23 (1.45-2.31)
Re-exploration	382/4,915	7.8%	5. 36 (2.54-10.09)
Need for IABP	547/3,717	14.7%	10. 25 (2.12-3.76)
Respiratory complications	403/2,257	17.86%	16.45 (4.86-43.13]
Length if ICU stay (days)		3.97	
Length of hospital stay (days)		8.95	
Post-op LVEF		53.22	

Discussion

The aim of this review was to review available data on outcomes following CABG surgery in the African continent. The review demonstrated that the post-operative outcomes following CABG in Africa are comparable to the results found in other regions. The majority of patients who underwent CABG in our study were males, similar to other studies [53-56].

In a multicentre study in North and South America, Europe, the Middle East, and Asia on patients who underwent CABG surgery, 67.5% had a history of hypertension; this is similar to the findings in our study that showed 64.2% of patients with hypertension [57]. Another study in Japan showed a pre-CABG hypertension proportion of 75.2% [58]. Marui et al. in their study reported that over 70% of their patients who underwent intervention for severe coronary artery disease had preserved LVEF with LVEF >50% [59], which is lower than that found in our study, reporting over 90% of the study participants with LVEF of >40%.

The proportion of patients with diabetes mellitus was much higher in our study at 45% compared to the study by Maria et al., which reported 30% of patients undergoing CABG having diabetes mellitus [57]. In 2022, the World Health Organisation reported Africa to be the leading continent with undiagnosed diabetes mellitus [60]. Diabetes mellitus is a known risk factor for developing coronary diseases, with uncontrolled diabetes mellitus posing a more significant risk [61,62]. Results from our study show that a higher proportion of patients undergoing CABG have DM in Africa compared to other nations. The high burden of undiagnosed DM, delayed diagnosis, and poor control of diagnosed DM in Africa [63] can serve as a strong risk factor for having severe coronary artery disease requiring surgical intervention in the continent.

Paez et al. in their population study in Brazil in 2019, with a sample size of over 2,000, reported an operative mortality of 2.8% and an incidence of stroke of 1.2% [64]. Our study produced a pooled mortality of 3.73%, which is higher than that of the 2.8% seen in the Brazilian population. The mortality rate is also slightly higher than that reported by Hawkins et al. whose review reported a conclusive overall mortality of about 2-3% following CABG [54]. In the United Kingdom (UK), the national audit reported the mortality rate of elective CABG was low (at 0.43% in 2021/2022) [65].

The pooled early mortality rate result of 3.51% obtained from our meta-analysis is higher than those found in other studies. In 2017, a 30-day mortality of 1.3% was reported in Canada [66]. Mediratta et al. in their UK study reported an in-hospital mortality post-CABG of 1.1% [55]. However, this rate can vary depending on the region in the UK [67]. In America, the Society of Thoracic Surgery reported a 30-day mortality rate of 2% following CABG [68]. These countries report much lower early mortality following CABG. A study by Jose et al. in India reported an early mortality rate of 11.62% [56]. This rate is higher than that reported in our meta-analysis. In their study, this high early mortality was explained by the increased proportion of salvage and emergency CABG surgeries performed at a primary referral centre [56]. Adelborg et al. in 2017 reported the outcomes of CABG in over 50,000 individuals in the Danish population over a 30-year period [53]. They reported a 30-day mortality of 3.2%, which is comparable to our study, which reported a pooled early mortality of 3.73%. This varied rate of in-hospital mortality following CABG across countries and regions might be related to operative technique. The socio-economic status of a country has also been found to contribute to post-operative outcomes, with countries with lower socio-economic status having poorer outcomes compared to developed countries.

The rate of surgical site infection in our study was 4.59%, which varied from those found in some studies. In a study done in the United States of America, a 1.1% incidence rate of sternal wound infection was reported [69]. This rate is much lower than that reported in our study. Another study done in Jordan, a middle-income country, reported a post-CABG surgical site infection rate of 16.8% [70], which is much higher than that reported in our systematic review of the African population. Studies have shown that the rate of surgical site infection is higher in low- and middle-income countries compared to high-income countries [71-73].

A post-CABG rate of atrial fibrillation was reported at 21.5% in the study by Magee et al. in the United States of America [74]; this is higher than that of our study, which showed a post-CABG atrial fibrillation rate of 13.9%. It has been reported that black ethnicity had a lower prevalence of atrial fibrillation than white ethnicity [75]. This could explain the lower proportion of patients with post-operative atrial fibrillation in our study compared to other populations.

Though pioneering cardiac surgery in Africa began in the 1950s with the first-ever heart transplant occurring in South Africa, many African countries, especially in sub-Saharan Africa, have a gross lack of access to cardiac surgery [76]. Forcillo et al. in 2019 identified several factors contributing to the lack of sustainable cardiac surgery centres and programs in Africa [77]. Cardiac surgeries are relatively expensive, requiring specialised equipment and devices. With the poor socio-economic status of many African countries, there is a lack of adequate funds to create new cardiac centres and to effectively sustain already established centres [1,10,77]. African countries continue to suffer devastating cuts in funds for healthcare [10]. In addition to this, Yankah et al. in their paper reported an incidence of one surgeon per 1.3 million people in Africa based on the number of registered cardiothoracic surgeons. This was worse in Sub-Saharan Africa, with a ratio of one surgeon per 3.3 million people [76]. They also reported an incidence of 12 cardiac surgeries per million persons in Africa. This lack of personnel, resources, and infrastructure in African countries could contribute to poorer outcomes following cardiac surgery.

Limitations of the Study

One major limitation of this review was the lack of adequate published original research papers from many other African countries. Coupled with this, some of the papers identified also had incomplete data and information needed to carry out a more extensive review. Though several case reports and case series were identified in our literature search in many other African countries, there were no identified papers that reported on either observational or controlled studies for population groups. Though these case reports could serve as a valuable addition to the body of research, they provide a low level of evidence in research as they do not represent the whole population [78].

Another limitation of the study was the exclusion of papers not written in English. These excluded papers could have provided relevant information on CABG outcomes in parts of Africa that are not English speaking. Moreover, the lack of access to a few papers served as a limitation. The authors lacked resources for interpretation of the non-English papers and purchasing papers without access to full text are limitations.

In addition, publication bias is another identified limitation of this study. It is possible that many unpublished studies may have been undertaken in other parts of Africa, and the reports from these unpublished data may provide very important information for our meta-analysis.

Recommendations

The lack of adequate research publications in African countries needs to be adequately addressed and the government needs to take steps to fund original research in many African countries. This would encourage clinicians to publish their outcomes so that factors that may be affecting poorer outcomes can be adequately identified and addressed.

## Conclusions

There are limited studies reporting outcomes following cardiac surgeries, especially CABG in many parts of Africa. This can be attributed to the lack of access to cardiac health in these parts, due to low socio-economic status and lack of funds. Despite this, the high number of case reports suggests that these cardiac surgeries were performed. It is, therefore, important that the available cardiac centres in Africa take steps to report on their surgical short-term and long-term outcomes. This would help researchers and clinicians to better understand factors affecting post-operative outcomes following CABG in this part of the world and how steps can be taken to improve these outcomes.
